# Supplementary material for: Aerosol Inhalation of Chimpanzee Adenovirus Vectors (ChAd68) Expressing Ancestral or Omicron BA.1 Stabilized Pre–Fusion Spike Glycoproteins Protects Non–Human Primates against SARS-CoV-2 Infection
Source: Vaccines (Basel). 2023 Aug 28;11(9):1427. doi: 10.3390/vaccines11091427 (PMC10535855; doi:10.3390/vaccines11091427)
Supplement: Supplementary file 1 [file vaccines-11-01427-s001.zip › vaccines-2524001-supplementary.pdf]

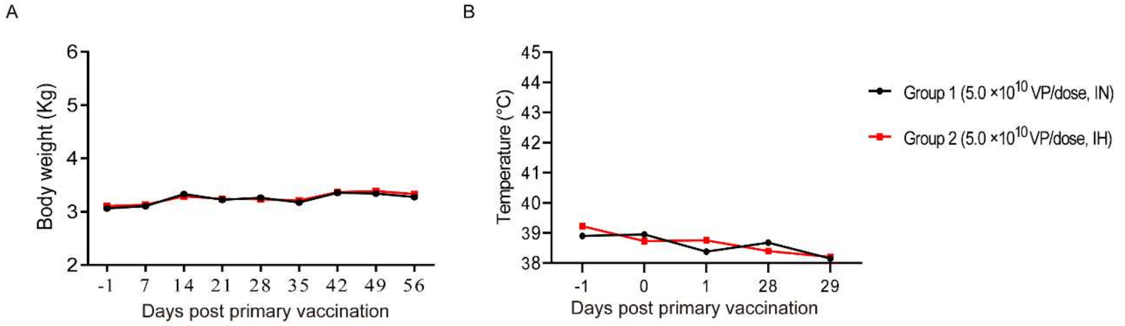

Figure S1. Bodyweight (A) and temperature (B) of cynomolgus monkeys after vaccination via the intranasal route (IN) or inhalation (IH). (A) Bodyweight of cynomolgus monkeys recorded every 7 days. (B) Body temperature of cynomolgus monkeys measured one-day pre-vaccination (D-1) on D0, D1, D28, and D29 after primary immunization.

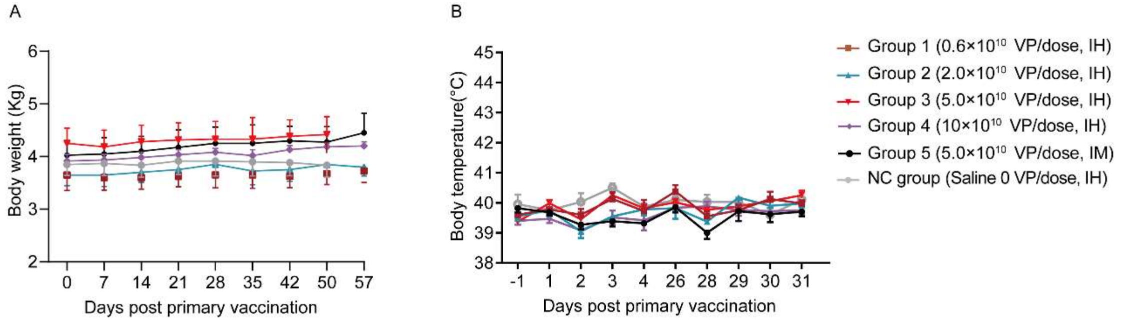

Figure S2. Body weight (A) and temperature (B) of rhesus macaques. (A) Body weight of rhesus macaques recorded every 7 days. (B) Body temperature of rhesus macaques measured on one-day pre-vaccination (D-1), D1, D2, D3, D4, D26, D28, D29, D30, and D31 after primary immunization.

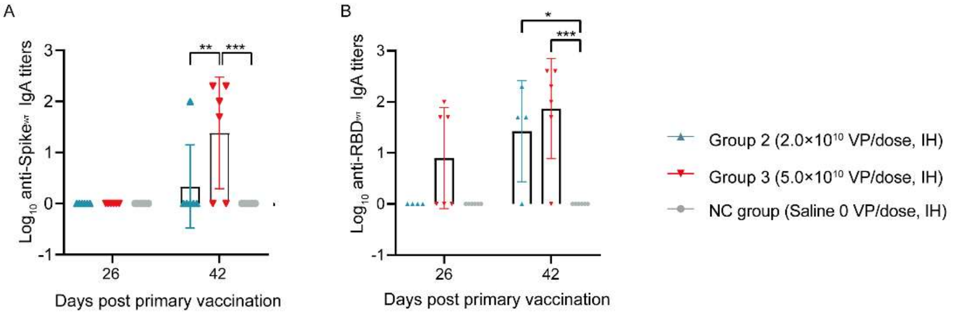

Figure S3. Serum anti-S and anti-RBD IgA antibody responses against the WT strain in rhesus macaques assessed by indirect ELISA. Monkeys in Group 2, Group 3, and NC group were vaccinated by aerosol inhalation of BV-AdCoV-1 or saline. (A) Serum anti-Spike IgA titers against the WT strain on D26 and D56 were determined by indirect ELISA. (B) Serum anti-RBD IgA titers against the WT strain on D26 and D56 were determined by indirect ELISA. Data are presented as means ± SD. The lower limit of detection is an IgA titer of 20 in sera. When the value is below 20, it is calculated as 1. Statistical analyses were performed using two-way ANOVA and Tukey's multiple comparison test. *$p<0.05$, **$p<0.01$, ***$p<0.001$.

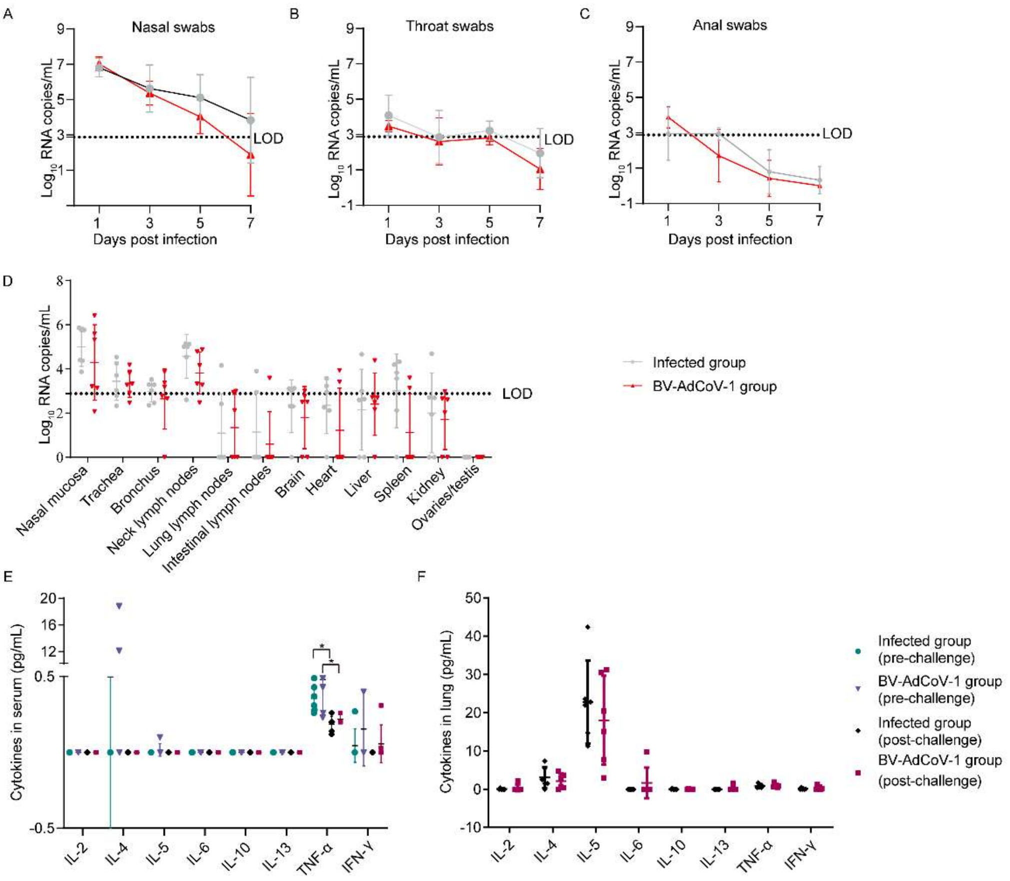

Figure S4. Viral load in rhesus macaques' tissues and cytokine levels in rhesus macaques' sera and lung homogenates. Rhesus macaques (n=6/group) received two doses of aerosolized BV-AdCoV-1 or saline and were then intratracheally and intranasally challenged with 1×10^6 pfu of live SARS-CoV-2 virus (GD108# strain which is similar to the Wuhan-Hu-1 strain) on D25 post-vaccination. Tissue or serum samples were collected after euthanasia. (A) RT-qPCR analysis of viral load in nasal swabs. (B) 1- RT-qPCR analysis of viral load in throat swabs. (C) RT-qPCR analysis of viral load in anal swabs. (D) RT-qPCR analysis of viral load in a panel of the tissue/organ homogenates. (E) Luminex analysis of cytokines expression in rhesus macaques' sera. (F) Luminex analysis of cytokines expression in rhesus macaques' lung homogenate. Data are presented as means ± SD. The dotted lines represent the limit of detection (LOD). Statistical analysis was performed using two-way ANOVA. The viral load of LOD is 2.88 log 10 copies/mL. *$p<0.05$.

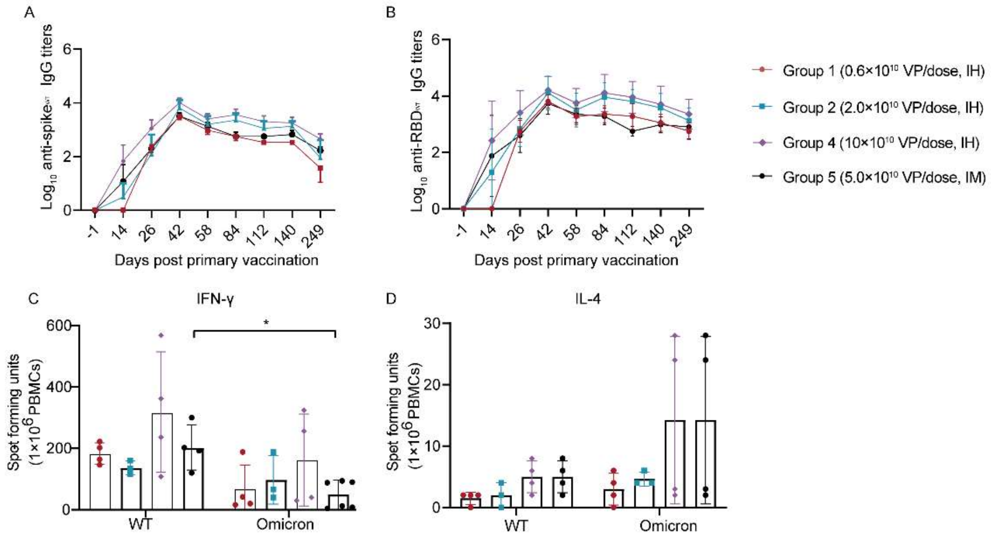

Figure S5. Serum anti-Spike and anti-RBD IgG antibody titers against wild-type SARS-CoV-2 in 18 rhesus macaques. Serum anti-Spike IgG titers in rhesus macaques in groups 1, 2, 4, and 5 were determined by indirect ELISA. (A) Serum-binding IgG antibody titers against S of wild-type SARS-CoV-2 in rhesus macaques in groups 1, 2, 4, and 5 were determined by indirect ELISA. (B) Serum-binding IgG antibody titers against RBD of wild-type SARS-CoV-2 strain in rhesus macaques in groups 1, 2, 4, and 5 were determined by indirect ELISA. (C) Numbers of IFN-γ-secreting PBMCs after WT or Omicron spike peptides pool stimulation were assessed by ELISpot assays on D283. (D) Numbers of IL-4-secreting PBMCs after WT or Omicron spike peptides pool stimulation were tested by ELISpot assays on D283. Statistical analysis was 2-way ANOVA and Tukey's multiple comparison test. *$p<0.05$.

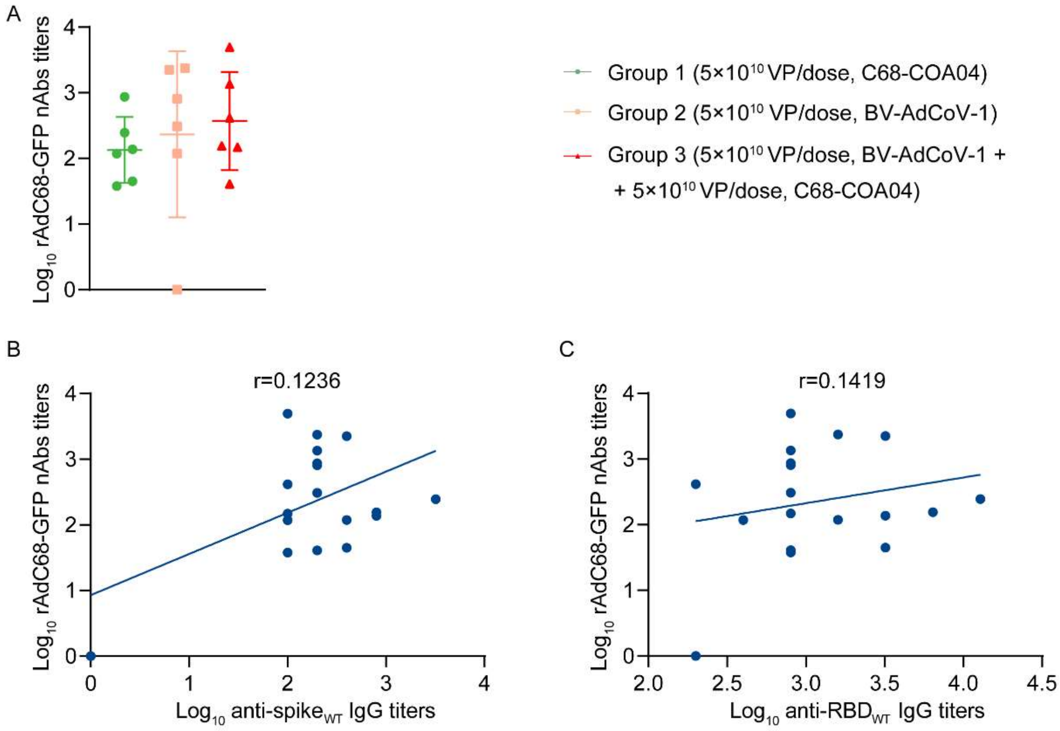

Figure S6. Anti-chimpanzee vector (rAdC68-GFP) neutralizing antibody titers in 18 rhesus macaques on D249. (A) Anti-rAdC68-GFP neutralizing levels in 18 rhesus macaques after regrouping on D249. The lower limit of detection is a pseudovirus-neutralizing antibody titer of 20 in sera. When the value is below 20, it is calculated as 1. (B) Correlation analysis of anti-rAdC68-GFP neutralizing levels and binding IgG antibody titers against S of wild-type SARS-CoV-2 on D249. (C) Correlation analysis of rAdC68-GFP neutralizing levels and binding IgG antibody titers against RBD of wild-type SARS-CoV-2 on D249. Statistical analysis was performed using a two-sided Spearman rank correlation.

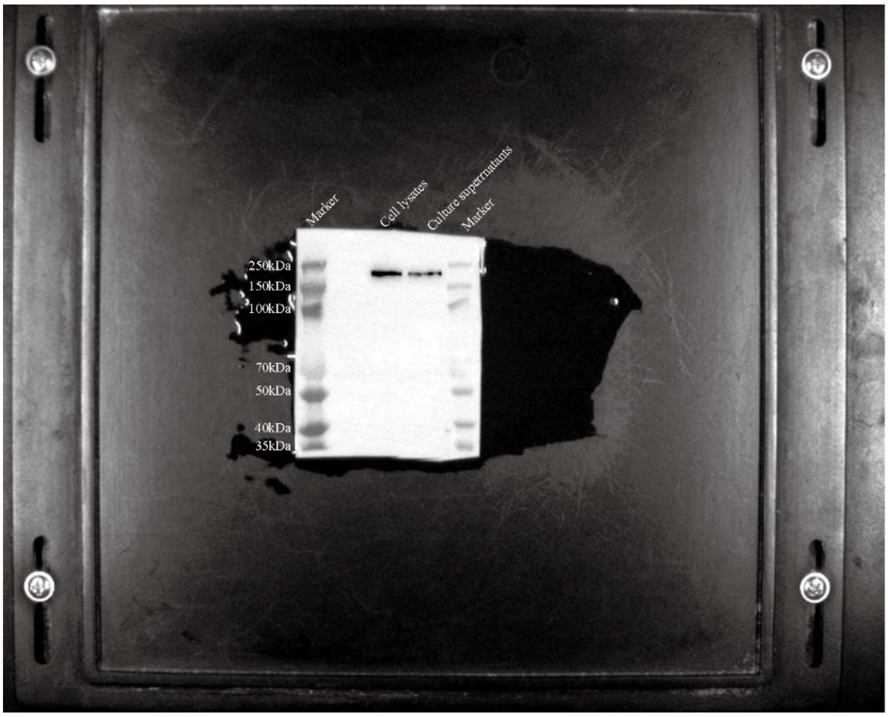

Figure S7. Western blot analysis of the whole blot (uncropped blots).

Table S1. Detailed grouping of 30 rhesus macaques.

Group	Vaccine	No. of Animals	Mode of Vaccination	Dosage
NC	Saline	6	inhalation (IH)	0 VP/dose
1	BV-AdCoV-1	4	inhalation (IH)	0.6×10^{10} VP/dose
2	BV-AdCoV-1	4	inhalation (IH)	2×10^{10} VP/dose
3	BV-AdCoV-1	6	inhalation (IH)	5×10^{10} VP /dose
4	BV-AdCoV-1	6	inhalation (IH)	10×10^{10} VP /dose
5	BV-AdCoV-1	4	intramuscular (IM)	5×10^{10} VP /dose

Table S2. Primer sequences for SARS-CoV-2 fluorescent RT-qPCR assay.

Primers	COVID-19 E_Sarbeco-F	5′-3′ ACAGGTACGTTAATAGTTAATAGCGT
	COVID-19 E_Sarbeco-R	5′-3′ ATATTGCAGCAGTACGCACACA
	Probe	5′-3′ ACACTAGCCATCCTTACTGCGCTTCG

Table S3. Post-challenge lung pathology scores of infected and vaccinated rhesus macaques. The scores for interstitial pneumonia and hemorrhage in lung tissues are listed in each column.

Groups	Animal ID	Interstitial Pneumonia	Hemorrhage	Total Score
Infected group	1001	0.125	1.875	2
	1002	1.375	0.625	2
	1003	1.5	1.375	2.875
	2004	0.625	2.375	3
	2005	3.625	0.125	3.75
	2006	2.5	2.25	4.75
BV-AdCoV-1 group	1315	0.625	1.875	2.5
	1316	0	3.125	3.125
	1317	0.375	1.875	2.25
	2318	0.75	2	2.75
	2319	0.625	0.625	1.25
	2320	2.75	0.5	3.25
